# MVBeetle: an interpretable multi-view deep learning model for fine-grained classification of Galerucinae and Alticinae (Coleoptera: Chrysomelidae)

**DOI:** 10.3389/fpls.2026.1798135

**Published:** 2026-04-07

**Authors:** Junhui Liu, Xiaoling Lin, Hao Shi, Si Li, Shiyu Li, Fangrong Liu, Yong Cao, Youjie Zhao, Ruie Nie

**Affiliations:** 1 College of Big Data and Intelligent Engineering, Southwest Forestry University, KunMing, China; 2 The Anhui Provincial Key Laboratory of Biodiversity Conservation and Ecological Security in the Yangtze River Basin, College of Life Sciences, Anhui Normal University, Wuhu, Anhui, China

**Keywords:** attention mechanism, chrysomelidae, classification, deep learning, multi-view

## Abstract

Galerucinae and Alticinae are typical herbivorous pests that seriously harm the growth of crops, trees, fruits, vegetables, and grasses worldwide. Historically, Galerucinae and Alticinae were treated as distinct sister subfamilies within Chrysomelidae. However, recent molecular phylogenetics and morphological reassessments have challenged this dichotomy, revealing that the boundary between these lineages is phylogenetically ambiguous. This study provides a systematic multi-view fusion framework, termed MVBeetle, for the convenient identification of the two target subfamilies. To this end, a multi-view image dataset was constructed based on synchronized high-resolution dorsal, lateral, and ventral views. The dataset comprises a total of 43 chrysomelid species, including 23 species from Galerucinae and 20 species from Alticinae. Subsequently, four convolutional neural network backbones (ResNet18, ResNet50, VGG16, and MobileNetV2) were developed as the core of MVBeetle by integrating multi-view features of leaf beetles. The experimental results show that the accuracy of multi-view fusion improved by 2.48%–12.95% compared with baseline models across the four networks. The optimized MVBeetle architecture achieved a peak classification accuracy of 94.44% ± 0.41%. Furthermore, Grad-CAM interpretability analysis indicated that MVBeetle’s attention significantly focused on key morphological features of different subfamilies (Alticinae and Galerucinae). Among these, the activation regions for Alticinae are mainly concentrated on the jumping legs, while Galerucinae focuses on the antennae. Importantly, cross-subfamily misclassifications were nearly zero, demonstrating the model’s strong taxonomic reliability. This study not only provides a high-precision and convenient classification model for leaf beetles, but also provides insights into the evolutionary morphology of beetles.

## 1 Introduction

Coleoptera represents the most species-rich insect order, accounting for approximately one-quarter of global animal diversity (Zhang et al., 2018). Within this hyper-diverse group, the family Chrysomelidae (also known as leaf beetles) constitutes a major evolutionary lineage with over 35,000 species (Jolivet, 2015). The majority of these taxa are significant herbivores that cause substantial economic losses to agricultural and forestry systems (Sánchez-Reyes et al., 2019). The subfamilies Galerucinae and Alticinae (flea beetles) constitute one of the most species-rich and ecologically complex groups within the family Chrysomelidae. Historically, taxonomists classified these groups as two distinct sister subfamilies based primarily on hind leg morphology and associated modes of locomotion. The traditional view holds that the diagnostic characteristic of the Alticinae is the significantly enlarged hind femora, which contain a specialized chitinous structure—the metafemoral spring (Maulik’s organ)—that confers a powerful jumping ability (Furth, 1988; Nadein and Betz, 2016). In contrast, the Galerucinae possess slender hind femora, lack the metafemoral spring, and are incapable of jumping. This dichotomy, based on a single functional morphological trait, dominated early insect classification systems (Mactlik, 1929; Gressitt and Kimoto, 1962).

With the development of molecular systematics, this traditional taxonomic boundary has faced severe challenges. Multiple studies based on nuclear genes and mitochondrial genomes consistently indicate that the traditionally defined “Galerucinae” is a paraphyletic group, whereas the “Alticinae” originated from within the Galerucinae lineage, representing a derived clade (Gómez-Zurita et al., 2007). The current phylogenetic consensus resolves this by treating them as distinct lingeages within a broader classification. Specifically, Galerucinae *sensu stricto* (tribe Galerucini) and Alticinae (tribe Alticini) are now recognized as sister groups, together constituting the subfamily Galerucinae *sensu lato* (Bouchard et al., 2011; Nie et al., 2018; Douglas et al., 2023). Despite this phylogenetic resolution, practical specimen examination remains impeded by the existence of numerous morphologically intermediate “problematic genera”. For instance, certain genera that cluster within the Alticini clade in molecular trees have secondarily lost the metafemoral spring and the associated jumping ability, while others exhibit conflicting character states. This high degree of morphological homoplasy and evolutionary complexity renders delimitation based solely on external morphology or traditional microscopic features highly controversial and difficult. Furthermore, while DNA-based methods offer high identification accuracy, their routine application in large-scale taxonomic research is constrained by practical limitations, including sequencing costs and laboratory requirements. Consequently, macroscopic morphology remains the most rapid and accessible basis for beetle classification. However, the transition to automated identification faces significant challenges; specifically, approaches based on single-view imagery have demonstrated limited effectiveness for fine-grained discrimination. This limitation arises because robust insect taxonomy typically necessitates the integration of multiple morphological characters rather than reliance on isolated visual information (Wen and Guyer, 2012; Engel and Kristensen, 2013).

With the development of artificial intelligence (AI), machine learning and deep learning techniques have been widely applied in insect classification (Teixeira et al., 2023). Early studies has shown that most automated approaches relied on handcrafted visual features combined with conventional machine learning classifiers, such as support vector machines and sparse representation-based models, which often showed limited adaptability under complex imaging conditions (Höferlin et al., 2012; Rather et al., 2017). Recent studies has shown that deep learning greatly improved insect identification performance through convolutional neural networks (CNNs) and vision transformers (ViTs) (Wäldchen and Mäder, 2018; Thenmozhi and Srinivasulu Reddy, 2019). Especially, deep learning models with attention mechanisms can help us find localization that distinguishes morphological cues (Guo et al., 2022). However, the majority of existing methods remain constrained to single-view image analysis (Woo et al., 2018; Wäldchen and Mäder, 2018; Wu et al., 2019). Such representations are insufficient to capture diagnostic traits distributed across different anatomical planes, highlighting the need for integrating complementary information from multiple viewpoints (Cardim Ferreira Lima et al., 2020; Høye et al., 2021; An et al., 2023).This limitation is especially evident in the closely subfamilies Alticinae and Galerucinae, where subtle morphological differences, considerable intraspecific variation, and environmentally influenced phenotypic plasticity complicate reliable discrimination under variable imaging conditions (Konstantinov, 1994; Battisti et al., 2005; Klink et al., 2022).

In this work, we establish the first multi-view image dataset for 43 species of Chrysomelidae, comprising 3,342 images captured from dorsal, ventral, and lateral perspectives. Based on this dataset, we proposed an interpretable multi-view deep learning model (MVBeetle) that integrates complementary morphological information for fine-grained taxonomic discrimination. Multiple convolutional neural network architectures are systematically evaluated under multi-view settings to assess the contribution of spatial feature fusion. In addition, Grad-CAM is employed to visualize model attention and relate salient regions to biologically meaningful morphological traits. The experimental results show that MVBeetle has higher classification performance and better biological interpretability. In short, this study provides a high-precision and interpretable classification model for leaf beetles.

## 2 Materials and methods

### 2.1 Dataset construction

#### 2.1.1 Specimen collection and digitization

In this work, we selected a total of 43 species for image acquisition, including 20 species from Alticinae and 23 from Galerucinae. To expand taxonomic coverage and morphological diversity, we additionally downloaded 1,546 high-quality images from the Global Biodiversity Information Facility (GBIF), which cover not only historical specimens deposited in the National Zoological Museum of China, the Natural History Museum (London), and the Smithsonian Institution but also a small portion of field-captured images (*in-situ*). We combined these GBIF and field images with 1,796 original high-resolution images acquired in this study through standardized three-view macro-photography. The specific imaging conditions and camera settings utilized for these original captures are detailed in **Supplementary Table 1**. This integration resulted in a comprehensive dataset of 3,342 images.

The dataset comprises three standardized viewpoints reflecting key morphological features used in leaf beetle taxonomy. The dorsal view shows the head, pronotum, elytra, antennal structure, compound eyes, pronotal shape, and dorsal surfaces of the legs. The lateral view provides a side profile, highlighting the head, thorax, abdomen, antennal orientation, and leg articulation. The ventral view captures the underside, including abdominal ventrites, leg attachment points, and the ventral surfaces of the antennae. Because diagnostically informative traits are distributed across all three perspectives, no single view alone is sufficient for accurate identification. Consequently, the MVBeetle model integrates all three views to reduce ambiguities inherent in single-view analysis and ensure robust morphological assessment.

#### 2.1.2 Morphology-preserving data augmentation

A multi-level data augmentation strategy was applied to increase the number of images per species and improve model generalization while preserving key morphological traits, such as hind femur proportions, antennal segments, and abdominal texture. Several operations were used to simulate real-world observation conditions, such as variations in viewing angle, uneven illumination, and minor camera movement. All augmentations were applied consistently across the dorsal, lateral, and ventral views of each specimen. All images were resized to 224 × 224 pixels using proportional scaling with black padding to preserve the original aspect ratio. The specific augmentation parameters and their corresponding biological and technical rationales are summarized in **Supplementary Table 2**.

#### 2.1.3 Dataset partitioning and significance

Ensuring the reproducibility of experimental results was our primary consideration; therefore, we partitioned the dataset using a single fixed stratified splitting strategy based on species-level distribution and physically separated it into independent training (70%), validation (15%), and testing (15%) directories rather than re-shuffling images across multiple experimental runs. The partitioning was implemented at the image level to maintain a consistent taxonomic distribution across all subsets. To ensure data independence, rigorous manual screening was conducted to strictly exclude primary images of the same specimen from the test set; however, due to the inherent limitation of a relatively small sample size, a negligible number of augmented derivatives (e.g., subtle geometric transformations) from the same specimen may still appear across different subsets. Nevertheless, the risk of data leakage was minimized to the greatest extent possible, thereby preserving the objectivity of the model’s generalization evaluation. This multi-view framework provides a robust morphological basis for both high-accuracy identification and Grad-CAM-based visualization (Selvaraju et al., 2017).

### 2.2 Model architectures and backbone selection

This study systematically evaluates four prominent CNN architectures—ResNet18, ResNet50, VGG16, and MobileNetV2—to assess their efficacy in the fine-grained discrimination of Alticinae and Galerucinae. The selection of these models is based on their distinct architectural philosophies, ranging from residual learning to depthwise separable convolutions, as summarized in **Table 1**. The ResNet family utilizes identity shortcut connections to facilitate deep-layer optimization, providing a scalable model for morphological feature extraction (He et al., 2016). Alternatively, VGG16 maintains a high inductive bias toward local spatial textures through a homogeneous stack of convolutional kernels (Simonyan and Zisserman, 2015), making it exceptionally sensitive to fine-scale structures like elytral punctation. Furthermore, MobileNetV2 emphasizes parameter efficiency via depthwise separable convolutions, representing an optimal solution for resource-constrained edge-device deployment (Sandler et al., 2018). All architectures were trained under a unified optimization framework incorporating an early stopping mechanism and a step learning rate scheduler, with comprehensive hyperparameter settings detailed in **Supplementary Table 3**. To ensure the robustness and reproducibility of the findings, each model was trained and evaluated using multiple random seeds (42, 100, 2024, 7, 99), with experiments repeated across these seeds to mitigate the influence of stochastic initialization and training dynamics on classification performance.

**Table 1:** Quantitative summary and design philosophy of the evaluated CNN backbones.

Backbone architecture	Parameters (M)	FLOPs (G)	Core design principles	Optimal research context
ResNet18	11.7	1.8	Residual learning; Gradient-stable	Rapid prototyping & Baseline benchmarking
ResNet50	25.6	4.1	Bottleneck blocks; High-level abstraction	Performance-efficiency trade-off
VGG16	138.3	15.5	Stacked kernels; Texture-centric	High-resolution morphological feature extraction
MobileNetV2	3.5	0.3	Inverted residuals; Linear bottlenecks	Mobile and edge-computing deployment

#### 2.2.1 Baseline architectures

In this study, we implemented the selected backbone networks as baseline models to evaluate their ability to extract discriminative features from the multi-view beetle images. For the baseline configuration, the three anatomical views (dorsal, lateral, and ventral) were directly provided to a conventional CNN model without explicit multi-view feature extraction, shared-weight branches, or attention mechanisms. This design serves as a straightforward reference for assessing the benefits of the proposed multi-view architecture.

All images were subjected to standardized preprocessing, including resizing to 224 × 224 pixels and an online data augmentation pipeline designed to reduce overfitting. We employed transfer learning using ImageNet-1K pre-trained weights, freezing low-level layers while fine-tuning higher-level layers to capture taxonomically relevant patterns. For each model, we appended a Global Average Pooling (GAP) layer followed by a Dropout layer to mitigate feature co-adaptation, and a fully connected layer producing outputs for the 43 target species. Model training was performed using the Adam optimizer in conjunction with a StepLR learning rate scheduler.

### 2.3 Architecture of the MVBeetle model

**Figure 1:**
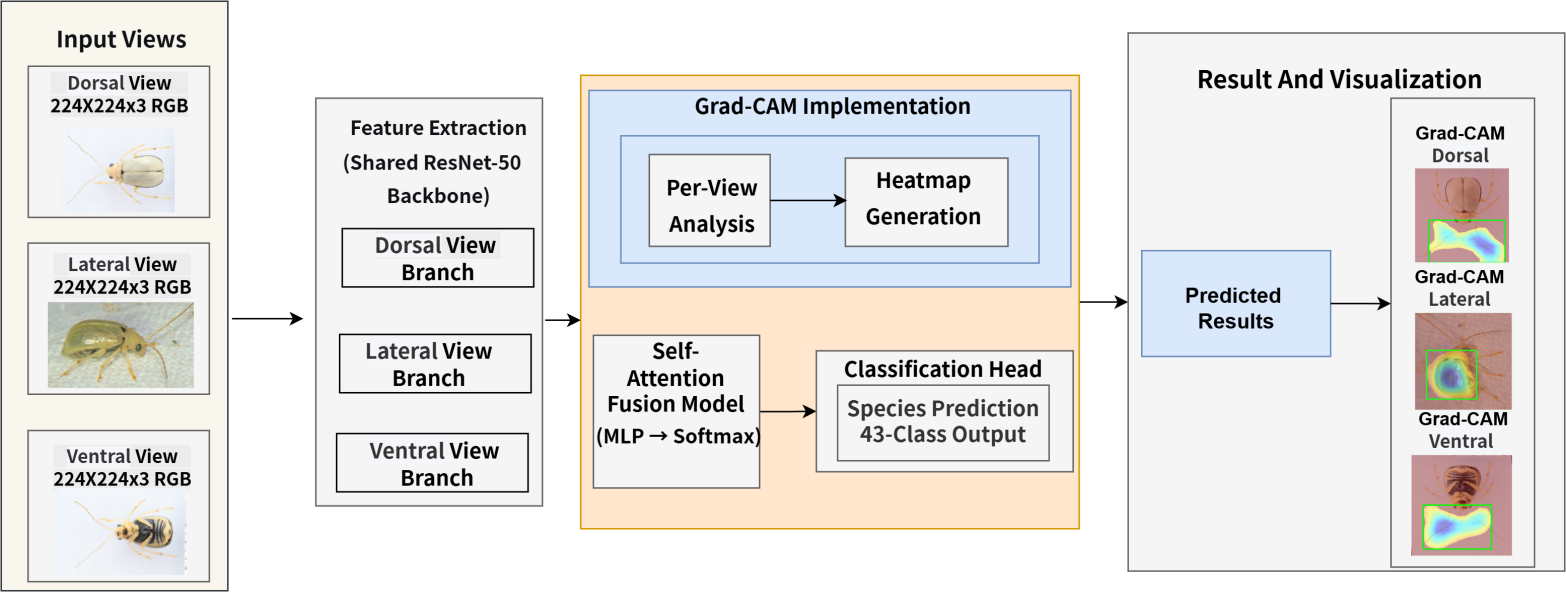
Overview of the proposed MVBeetle model architecture (ResNet50 shown as an example).

The MVBeetle model is specifically engineered for the fine-grained classification of Alticinae and Galerucinae. The core objective lies in fully exploiting the complementary morphological information provided by multiple insect perspectives, ensuring that disparate anatomical details are synthesized into a cohesive diagnostic representation. As illustrated in the global architecture (**Figure 1**), the model integrates three fundamental pillars: a Shared-Weight Feature Encoder, a Self-adaptive View Attention Fusion Module, and an Explainability Analysis Module driven by Grad-CAM.

This design ensures that the MVBeetle model maintains high taxonomical fidelity by processing dorsal, lateral, and ventral inputs through a unified parameter space. By employing a shared-weight strategy, the model captures consistent semantic features across different views while significantly reducing computational redundancy. Subsequently, the attention fusion module allows the model to dynamically prioritize the most informative anatomical regions, effectively mimicking the multi-angle diagnostic process used by human experts.

The initial phase involves the synchronous extraction of deep visual representations, where dorsal, lateral, and ventral images are processed in parallel through a shared convolutional backbone. This Shared-Weight Feature Encoder is pivotal for maintaining a consistent set of visual filters across all views, thereby capturing the specimen’s complex morphology with high fidelity. The empirical selection of this backbone is informed by the structural attributes and design philosophies detailed in **Table 1**, ensuring an optimal balance between computational efficiency and feature abstraction. Subsequently, these view-specific feature streams are funneled into a Self-adaptive View Attention Fusion Module. This attention-based mechanism performs dynamic prioritization by assigning adaptive weights to each perspective, allowing the network to emphasize the most informative anatomical landmarks—such as the saltatorial hind legs in the lateral view—while generating a unified multi-view descriptor for 43-class identification.

Simultaneously, the model incorporates an interpretability pipeline to bridge the gap between algorithmic outputs and entomological expertise. This analytical module leverages Grad-CAM to project the model’s attentional saliency onto the input images for each view. By visualizing the discriminative morphological regions that drive final predictions, the model provides a transparent verification of its decision-making process. Consequently, this ensures that the network successfully localizes biologically diagnostic traits, such as the hypertrophied hind femora, rather than converging on spurious background correlations.

#### 2.3.1 Shared-weight feature encoder

**Figure 2:**
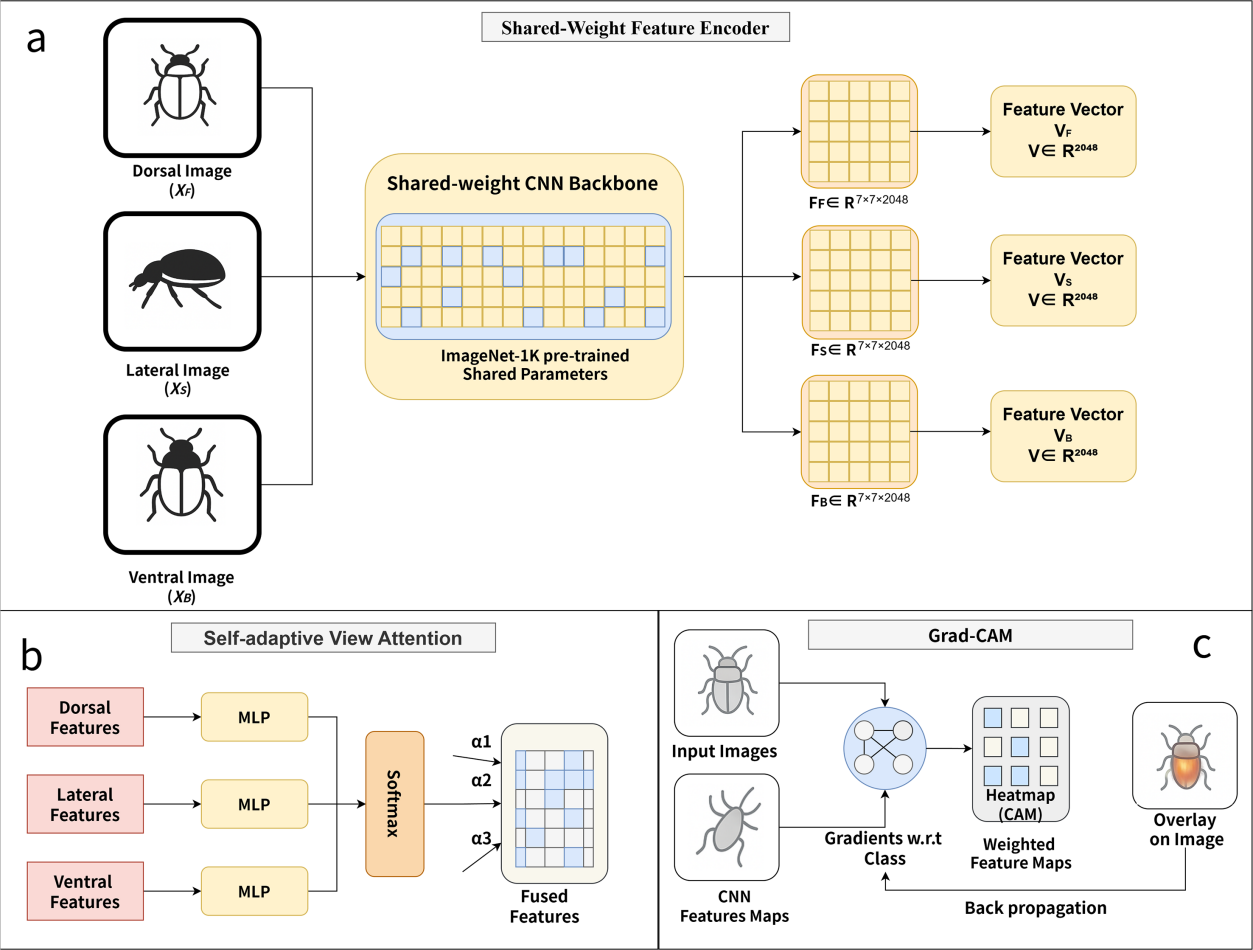
Workflow of MVBeetle feature encoding, attention fusion, and Grad-CAM explainability analysis. **(a)** Shared-Weight Feature Encode. **(b)** SVA, Self-adaptive View Attention. **(c)** Grad-CAM Visualization Process.

The shared-weight feature encoding strategy is illustrated in **Figure 2a**. To ensure representational consistency across different perspectives, the dorsal, lateral, and ventral images are all processed by the same convolutional backbone network, with parameters shared among views. Four CNN backbones—ResNet18, ResNet50, VGG16, and MobileNetV2—are evaluated in this model, all initialized with ImageNet-1K pre-trained weights.

Formally, for the v-th perspective image ${\text{I}}_{\text{v}}$(where v∈{dorsal,lateral,ventral}), the shared backbone extracts the final convolutional feature map as Equation 1:

(1) $$\begin{array}{c} F_{v}=f_{\theta }\left(I_{v}\right) \end{array}$$

where ${\text{f}}_{\text{θ}}(\cdot )$ denotes the shared convolutional feature extractor and ${\text{F}}_{\text{v}}$represents the output feature map from the last convolutional layer.

To ensure that only latent visual representations are retained, the original classification layer of each backbone is replaced with an identity mapping Equation 2:

(2) $$\begin{array}{c} backbone.fc=Identity() \end{array}$$

The resulting feature maps ${\text{F}}_{\text{v}}\in {\text{R}}^{7\times 7\times C}$(with C=2048 for ResNet50) are subsequently transformed into compact feature vectors using Global Average Pooling (GAP) Equation 3:

(3) $$\begin{array}{c} f_{v}=GAP\left(F_{v}\right) \end{array}$$

yielding a C-dimensional representation for each view. This design enables efficient encoding of morphological traits while maintaining architectural consistency across perspectives.

#### 2.3.2 Self-adaptive view attention fusion module

The Self-adaptive View Attention fusion mechanism is illustrated in **Figure 2b**. To dynamically model the relative importance of different views, a lightweight MLP-based scoring network is introduced to compute an attention score for each view-specific feature vector.

For each perspective, the importance score ${\text{S}}_{\text{v}}$is computed as Equation 4:

(4) $$\begin{array}{c} S_{v}=\sigma \left(W_{v}f_{v}+b_{v}\right) \end{array}$$

where ${\text{W}}_{\text{v}}$and ${\text{b}}_{\text{v}}$denote the learnable parameters of the scoring network, and σ(·) represents a nonlinear activation function. The scores from all views are then normalized using the Softmax function to obtain attention weights Equation 5:

(5) $$\begin{array}{c} \alpha_{v}=Softmax\left(S_{v}\right) \end{array}$$

The final fused multi-view feature vector is computed as a weighted summation of the individual view features Equation 6:

(6) $$\begin{array}{c} f_{fusion}=\underset{v}{\sum }\alpha_{v}f_{v} \end{array}$$

This attention-driven fusion strategy allows the network to emphasize the most discriminative perspectives for a given specimen, such as hind femoral enlargement in the lateral view, abdominal segmentation patterns in the ventral view, or antenna morphology in the dorsal view.

#### 2.3.3 Training strategy and optimization

The optimization process for the MVBeetle framework integrates a series of refined methodologies to ensure structural stability and robust generalization across the multi-species morphological dataset of beetles. All experiments were executed in a high-performance computing environment using a consistent hardware–software stack to ensure reproducible acceleration (detailed specifications are provided in **Supplementary Table 3**).

A differentiated layer unfreezing strategy was implemented to balance the retention of general-purpose visual filters with the necessity for biological specialization. The depth of fine-tuning was calibrated to the structural characteristics of each backbone: late-stage residual blocks were unfrozen for the ResNet series to capture interspecific structural variations; convolutional layers were fully unfrozen for VGG16 to maximize sensitivity to fine-scale local textures; while for MobileNetV2, only the final feature block was optimized to preserve its lightweight representation.

To enhance computational efficiency without compromising numerical precision, Automatic Mixed Precision (AMP) was applied throughout the learning phase. The learning objective was optimized using the Adam optimizer, integrated with a dynamic learning rate scheduling mechanism and a weight decay coefficient to prevent overfitting.

As specified in **Supplementary Table 3**, all hyperparameters were determined through a rigorous validation process. To ensure the statistical reliability of the performance metrics across the 43 target species, the training process was repeated using multiple random seeds using a set of fixed random seeds, with an early stopping criterion implemented to ensure optimal convergence.

#### 2.3.4 Performance evaluation metrics

Model performance is evaluated using multiple complementary metrics, including Top-1 and Top-5 accuracy, macro-averaged Precision, Recall, and F1-score, One-vs-Rest AUC, and species-level confusion matrices.

#### 2.3.5 Explainability via Grad-CAM

The Grad-CAM–based explainability pipeline is illustrated in **Figure 2c**. By applying Grad-CAM to its final convolutional feature maps, the MVBeetle model identifies class-discriminative regions to clarify its internal decision-making process. This approach generates spatial heatmaps that highlight the anatomical features most influential to the final classification, thereby ensuring the model’s outputs are both transparent and biologically grounded.

For a target class c, the channel-wise importance weights are computed as Equation 7:

(7) $$\begin{array}{c} \alpha_{k}^{c}=\frac{1}{Z}\underset{i}{\sum }\underset{j}{\sum }\frac{\partial y^{c}}{\partial A_{ij}^{k}} \end{array}$$

where ${\text{A}}^{\text{k}}$denotes the k-th feature map and Zis a normalization factor. The resulting Grad-CAM heatmap is obtained as Equation 8:

(8) $$\begin{array}{c} S^{c}=ReLU\left(\underset{k}{\sum }\alpha_{k}^{c}A^{k}\right) \end{array}$$

The generation of Class Activation Maps (CAM) involves upsampling the attention heatmaps to the original input resolution, followed by an overlay process to visualize the region most critical to the model’s prediction. A secondary post-processing pipeline is further applied to refine these visualizations, encompassing heatmap normalization, top-20% activation selection, and morphological refinement. Subsequent steps, including connected component analysis and bounding box generation, facilitate the precise anatomical mapping of these activated regions. Such a structured approach enables an explicit association between algorithmic focus and biologically diagnostic traits, such as hind femoral enlargement, abdominal segmentation textures, and antennal structures.

#### 2.3.6 Quantification of Grad-CAM activations

In this study, a statistical framework was further developed to quantitatively interpret the visual explanations generated by Grad-CAM. This methodology measures the frequency with which specific morphological regions are highlighted during model inference, effectively transforming qualitative heatmap visualizations into measurable indicators. These indicators reflect the model’s diagnostic focus across different insect subfamilies and imaging perspectives, providing a rigorous basis for taxonomic interpretability.

The Activation Rate is defined as the probability that a particular anatomical region is identified as a salient area in the Grad-CAM heatmap within a given subfamily–view subset. High-intensity hotspots corresponding to major morphological structures—including the head, thorax, abdomen, antennae, and legs—were examined to determine the dominant activation regions for each Grad-CAM visualization. This spatial analysis transforms qualitative heatmaps into quantifiable diagnostic indicators, providing a rigorous statistical basis for evaluating the model’s taxonomic focus across different insect groups.

If a single image simultaneously displayed strong activations across multiple anatomical regions (e.g., both the head and legs), each region was recorded independently to reflect the model’s multi-feature attention behavior. The activation rate for region r was then calculated as:

(9) $$\begin{array}{c} \text{Activation Rate}\left(r\right)=\frac{N_{r}}{N} \end{array}$$

where $N_{r}$ denotes the number of images in which region (r) exhibited significant activation and N represents the total number of images within the corresponding subfamily–view subset.

## 3 Results

### 3.1 Construction and characterization of the image dataset

**Figure 3:**
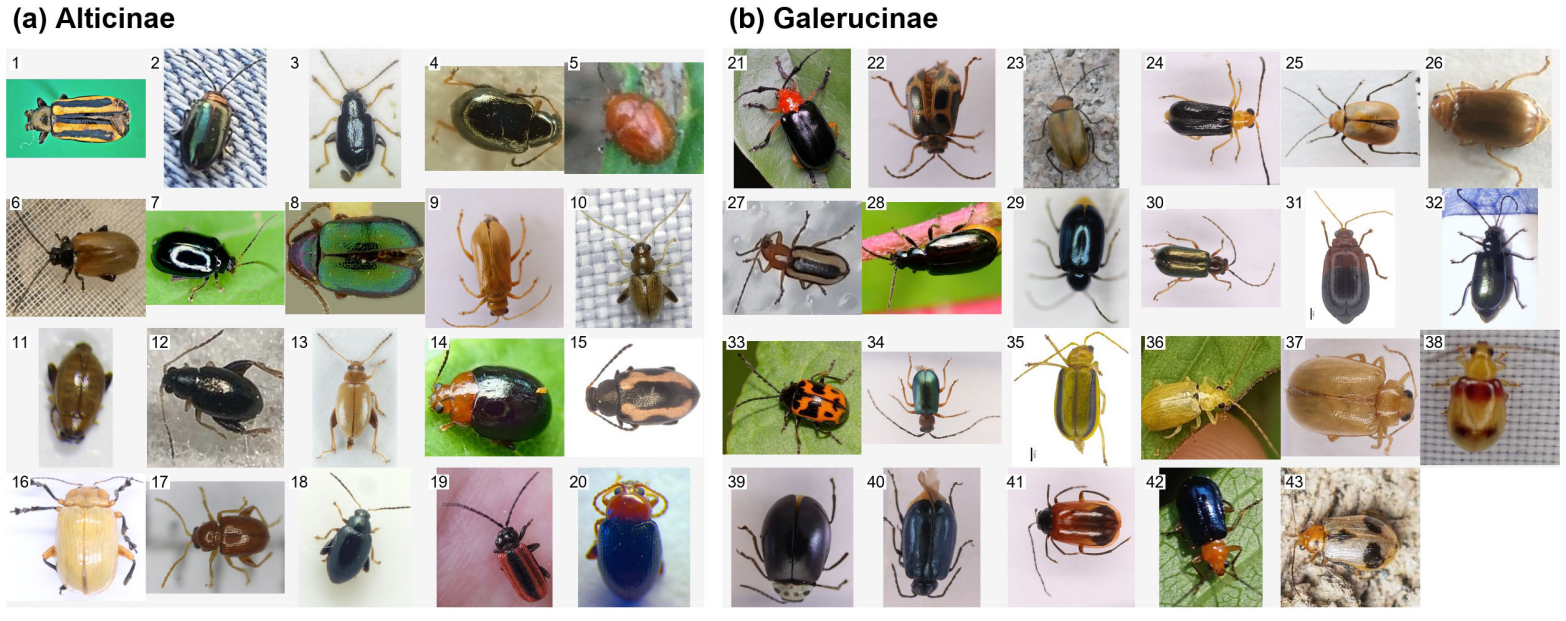
Examples of dorsal view images for the 43 investigated species. **(a)** Alticinae (Nos. 1–20) and **(b)** Galerucinae (Nos. 21–43).

**Figure 4:**
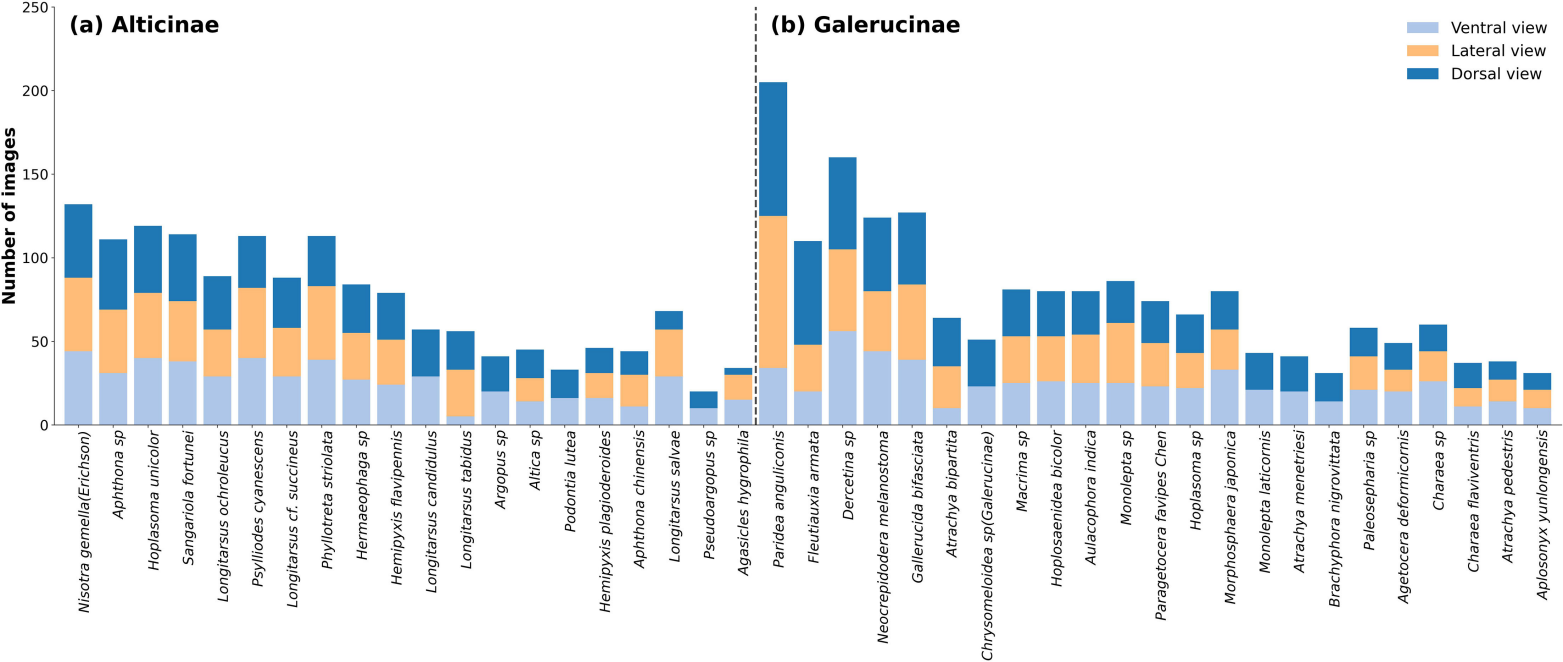
Species-wise distribution of multi-view images in **(a)** Alticinae and **(b)** Galerucinae.

In this study, we conducted a comprehensive statistical analysis of the constructed dataset to assess the effectiveness of the proposed preprocessing and dataset integration strategy across raw, external, and augmented multi-view samples. Representative specimens after preprocessing are illustrated in **Figure 3**, demonstrating the standardized dorsal orientation and consistent image quality achieved through the image normalization pipeline. **Figure 4** further presents the species-wise distribution of multi-view images in Alticinae (**Figure 4a**) and Galerucinae (**Figure 4b**), where stacked bars depict the relative contributions of ventral, lateral, and dorsal views for each species. Despite interspecific variation in sample size, most species show a comparatively balanced representation across views, and approximately 85% of the samples contain complete tri-view information, providing a solid foundation for subsequent multi-view feature fusion. The overall dataset composition and scale are summarized in **Supplementary Tables 4, S5**, including original macro-images, museum specimens curated from digitized GBIF records, and their augmented counterparts.

### 3.2 Species classification results

#### 3.2.1 Analysis and evaluation of experimental results

The comparative results summarized in **Table 2** illustrate a clear trajectory of performance gains achieved through the structural optimization of the MVBeetle framework. By evaluating three configurations—Baseline Model, Multi-View Concat (shared-weight encoding), and the full MVBeetle with attention-based multi-view fusion—a consistent, stepwise improvement in classification performance is observed across all four CNN backbones. The transition from the baseline model to the Multi-View Concat configuration underscores the benefit of spatial redundancy and shared-weight feature extraction. By processing dorsal, lateral, and ventral perspectives through a unified encoder, the models learn a consolidated feature representation that mitigates perspective-specific occlusions. For instance, in VGG16, this structural modification alone increases test accuracy from 81.49% ± 0.52% to 88.26% ± 1.33%, corresponding to a net gain of +6.77%, while ResNet18 achieves a +5.38% improvement in the concatenation mode. Even this simple multi-view integration provides a more robust foundation for insect identification compared with single-view approaches. Statistical analysis in **Supplementary Table 6** confirms that all observed performance improvements are significant: for ResNet18 and ResNet50, both McNemar, paired t-test, and Wilcoxon tests indicate highly significant gains (p< 0.01), while VGG16 and MobileNetV2 also show consistent improvements, with most comparisons reaching p< 0.05. These results demonstrate that multi-view integration consistently enhances classification performance across all four backbone networks.

**Table 2:** Ablation comparison of baseline model, multi-view (Concat), and attention-based MVBeetle across four CNN backbones (Mean (Std)).

Model	View mode	Acc (%)	F1	AUC	Precision	Recall	Top-5 (%)	Δ Acc
ResNet18	Baseline	82.99 (1.64)	0.818 (0.020)	0.9915 (0.0015)	0.835 (0.020)	0.805 (0.021)	96.32 (0.41)	—
	Concat	88.37 (1.43)	0.821 (0.026)	0.9890 (0.0002)	0.790 (0.028)	0.760 (0.030)	96.85 (0.60)	+5.38
	**Multi-View**	**92.70 (1.63)**	**0.900 (0.035)**	**0.9932 (0.0016)**	**0.915 (0.030)**	**0.895 (0.032)**	**98.60 (0.53)**	**+9.71**
ResNet50	Baseline	84.84 (1.98)	0.845 (0.017)	0.9947 (0.0013)	0.921 (0.020)	0.832 (0.020)	96.50 (0.50)	—
	Concat	89.05 (1.20)	0.861 (0.019)	0.9953 (0.0002)	0.872 (0.018)	0.918 (0.018)	97.62 (0.38)	+4.21
	**Multi-View**	**93.48 (0.57)**	**0.898 (0.020)**	**0.9921 (0.0040)**	**0.912 (0.020)**	**0.885 (0.022)**	**98.94 (0.26)**	**+8.64**
VGG16	Baseline	81.49 (0.52)	0.801 (0.005)	0.9922 (0.0003)	0.812 (0.008)	0.792 (0.009)	92.60 (0.60)	—
	Concat	88.26 (1.33)	0.879 (0.019)	0.9979 (0.0003)	0.894 (0.015)	0.897 (0.014)	97.75 (0.18)	+6.77
	**Multi-View**	**94.44 (0.41)**	**0.934 (0.010)**	**0.9996 (0.0002)**	**0.947 (0.008)**	**0.935 (0.013)**	**99.22 (0.35)**	**+12.95**
MobileNetV2	Baseline	87.80 (0.35)	0.806 (0.004)	0.9965 (0.0002)	0.816 (0.006)	0.826 (0.006)	96.60 (0.30)	—
	Concat	88.20 (0.87)	0.809 (0.010)	0.9979 (0.0003)	0.800 (0.012)	0.822 (0.010)	97.13 (0.36)	+0.40
	**Multi-View**	**90.28 (1.15)**	**0.864 (0.017)**	**0.9985 (0.0003)**	**0.876 (0.015)**	**0.855 (0.018)**	**98.09 (0.31)**	**+2.48**

^1^Bold values represent the performance of our model.

The most pronounced performance improvement occurs with the incorporation of the attention mechanism in the full MVBeetle model. Unlike the Multi-View Concat configuration, which treats all view features equally, attention-based fusion dynamically recalibrates feature weights according to their diagnostic relevance. This is exemplified by ResNet18, where the addition of attention increases accuracy from 88.37% to 92.70%, yielding an additional gain of +4.33% and a cumulative improvement of +9.71%. The attention module effectively emphasizes high-information morphological regions—such as the metafemoral spring in Alticinae—while suppressing non-informative or background features, accounting for the observed improvements in Macro F1-score and OvR AUC.

Among all backbones, VGG16 in the attention-based multi-view configuration reaches the highest performance, achieving a test accuracy of 94.44% ± 0.41% and an OvR AUC of 0.9996 ± 0.0002. Even the lightweight MobileNetV2 benefits from the attention-driven design, showing a +2.48% accuracy gain and a Top-5 Accuracy of 98.09%, demonstrating the framework’s applicability to resource-constrained environments. Beyond raw accuracy, the multi-view models exhibit enhanced stability and reliability: all attention-based configurations maintain Top-5 Accuracy above 98.09%, and a minimal difference between Precision and Recall (e.g., 0.012 for VGG16) indicates a substantial reduction in misclassification. This cross-view compensatory effect shows that MVBeetle does not simply aggregate visual inputs but leverages multi-perspective synergy to emulate expert taxonomic observation, ensuring high-fidelity classification even under suboptimal imaging conditions where certain diagnostic traits may be partially obscured.

#### 3.2.2 Dual-view ablation study

Ablation experiments were further extended to investigate the complementary interactions between view pairs. In the ablation study focusing on dual-view configurations (**Table 3**), the performance differences among various view combinations highlight the relative contribution of each perspective to species classification. Overall, the Dorsal + Ventral combination consistently achieves the highest accuracy across all backbone architectures, indicating that the integration of dorsal and ventral information provides the most complementary coverage of critical morphological features. For instance, ResNet50–MVBeetle attains 92.63% accuracy, while VGG16–MVBeetle reaches 93.17%, demonstrating that the dorsal-ventral combination effectively captures complex body proportions, dorsal patterns, and ventral textures. These results emphasize the crucial role of ventral information in fine-grained taxonomic identification.

**Table 3:** Dual-view ablation study performance (test accuracy, mean ± Std).

Backbone (configuration)	Dorsal + lateral	Dorsal + ventral	Lateral + ventral
ResNet18–Baseline	80.58 ± 1.80	85.40 ± 1.27	82.74 ± 2.02
ResNet18–MVBeetle	88.77 ± 1.64	92.01 ± 1.76	91.47 ± 1.04
ResNet50–Baseline	84.76 ± 1.48	89.41 ± 1.30	87.01 ± 1.96
ResNet50–MVBeetle	87.18 ± 2.52	92.63 ± 1.48	90.78 ± 1.31
VGG16–Baseline	79.46 ± 0.82	83.39 ± 0.22	82.70 ± 0.48
VGG16–MVBeetle	90.31 ± 0.94	93.17 ± 0.25	92.93 ± 0.97
MobileNetV2–Baseline	85.22 ± 0.50	87.05 ± 0.52	86.80 ± 0.50
MobileNetV2–MVBeetle	88.97 ± 1.21	92.16 ± 0.41	91.47 ± 0.99

The second-best-performing combination is Lateral + Ventral, which, although slightly lower than Dorsal + Ventral, still substantially outperforms the Dorsal + Lateral combination. This pairing provides depth information and detailed morphological cues from the side and ventral views, such as the junctions of the hind legs and abdominal structures in Alticinae, aiding the model in detecting diagnostic features. However, its slightly lower performance reflects the limited coverage of dorsal patterns and texture details compared with the dorsal-ventral combination.

The lowest-performing combination is Dorsal + Lateral, which, while capturing frontal and side spatial features, lacks detailed ventral information, leading to incomplete representation of key diagnostic regions such as the ventral leg structures and abdominal traits. As a result, accuracy for ResNet50–MVBeetle drops to 87.18%, and VGG16–MVBeetle only reaches 90.31%, underscoring the limitations of excluding ventral view information for fine-grained classification.

Notably, across all dual-view combinations, MVBeetle consistently outperforms the corresponding baseline models, highlighting the benefits of attention-based multi-view feature integration. For example, in ResNet18–MVBeetle, the Dorsal + Ventral combination improves accuracy from 85.40% to 92.01%, demonstrating the model’s ability to dynamically assign weights to complementary view features and effectively focus on critical morphological regions. This multi-view synergy reduces ambiguities caused by missing information in any single perspective, ensuring both high accuracy and robustness.

In summary, the dual-view ablation study validates the necessity of multi-perspective integration and highlights the advantages of the MVBeetle framework. Across all backbones, the performance ranking follows Dorsal + Ventral > Lateral + Ventral > Dorsal + Lateral, consistent with the model’s ability to optimally leverage complementary visual information. These findings provide empirical support for full three-view fusion and confirm that attention-driven multi-view learning substantially enhances feature extraction and classification performance in fine-grained beetle identification tasks.

#### 3.2.3 Confusion matrix results

**Figure 5:**
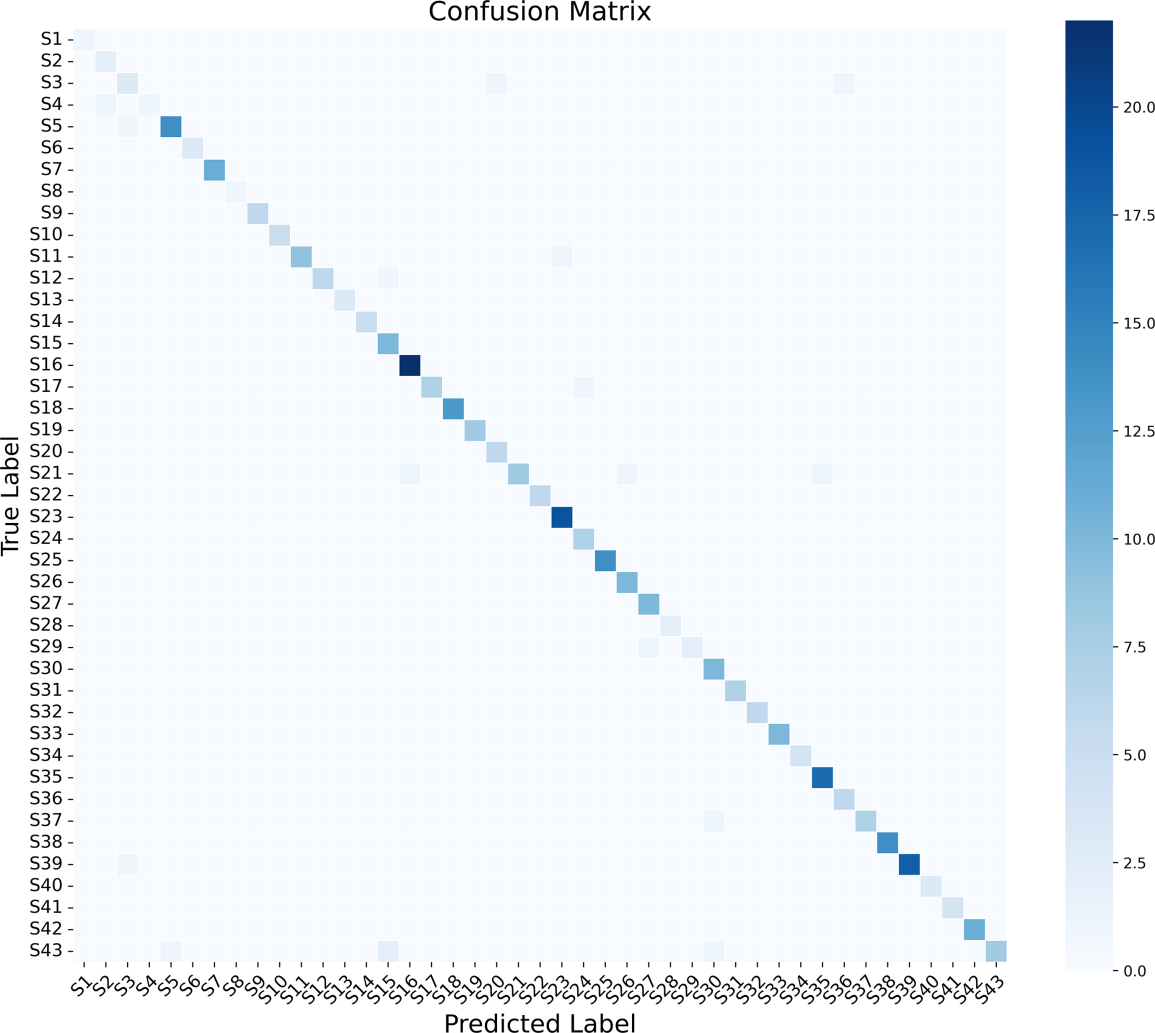
Normalized confusion matrix of the VGG16-based multi-view fusion model.

The normalized confusion matrix (**Figure 5**) provides a comprehensive evaluation of the species-level classification performance for the VGG16-based multi-view fusion model. Covering 43 Chrysomelidae species (labeled S1–S43; see **Supplementary Table 5** for the corresponding species names), the matrix directly illustrates the model’s robust discriminatory ability when confronted with fine-grained phenotypic variations. The diagonal dominance is immediately evident, indicating that the vast majority of predictions perfectly align with their corresponding ground-truth labels. Well-represented taxa, such as Dercetina sp. (S16), are classified almost flawlessly, while species with limited training data, such as *Aplosonyx yunlongensis* (S6), still achieve high accuracy.

Although overall misclassification is rare, a detailed examination of the off-diagonal elements reveals that the primary classification challenges are concentrated within intra-generic ambiguities. The most prominent example is observed within the genus Longitarsus, where S29 (*Longitarsus tabidus*) was occasionally misidentified as S2 (*Longitarsus ochroleucus*). This intrageneric confusion is primarily attributable to the extreme morphological convergence between these congeners, both of which feature near-identical pale ocherous coloration and ovate body profiles. Diagnostic differences—typically residing in microscopic elytral punctation and antennal segment ratios—are easily obscured during feature extraction or limited by image resolution. This suggests that while the MVBeetle framework excels at higher-level taxonomic separation, resolving such sibling species clusters may require the integration of higher-resolution local feature magnification or specialized attention to micro-morphological markers.

Furthermore, inter-generic morphological similarities within the same subfamily constitute a secondary source of error. Representative examples include the misclassification of S5 (Aphthona sp.) as S3 (*Altica* sp.), and S21 (*Hermaeophaga* sp.) as S35 (*Nisotra gemella*). These errors are rooted in the significant evolutionary convergence within the Alticinae subfamily, characterized by shared metallic integuments, globose body shapes, and similar limb proportions. For instance, the diagnostic hallmark of Hermaeophaga—the transverse basal impression on the pronotum—may be inadequately resolved or obscured by glare in certain views, leading the attention mechanism to prioritize dominant global features like elytral curvature, which biases the feature representation toward S35.

Interestingly, the misclassifications made by the model largely mirror the inherent difficulties faced by human taxonomic experts. Identifying Longitarsus species frequently requires microscopic examination of the aedeagus or internal sclerites, features that are inherently invisible in external morphological imaging.

Crucially, the model’s virtually no cross-subfamily misclassification demonstrates a level of stability that rivals expert judgment. While a human might occasionally overlook the metafemoral spring in a poorly angled single-view photograph, the MVBeetle’s integration of dorsal, lateral, and ventral perspectives ensures that this subfamilial diagnostic trait is consistently captured. This indicates that the model has successfully learned a “hierarchical” taxonomic logic: it first secures high-level subfamilial separation based on robust structural traits before attempting the more nuanced task of lower-level species differentiation.

### 3.3 Grad-CAM-based model interpretability

Our study utilizes Grad-CAM to visualize the model’s feature activations across Alticinae and Galerucinae samples. The primary objective is to uncover the underlying decision-making process of the deep learning architecture by observing its focus across multiple perspectives. By quantifying how often specific body parts are “activated,” this analysis confirms that the model relies on biologically meaningful traits—such as specialized leg structures—rather than being misled by random background noise.

**Figure 6:**
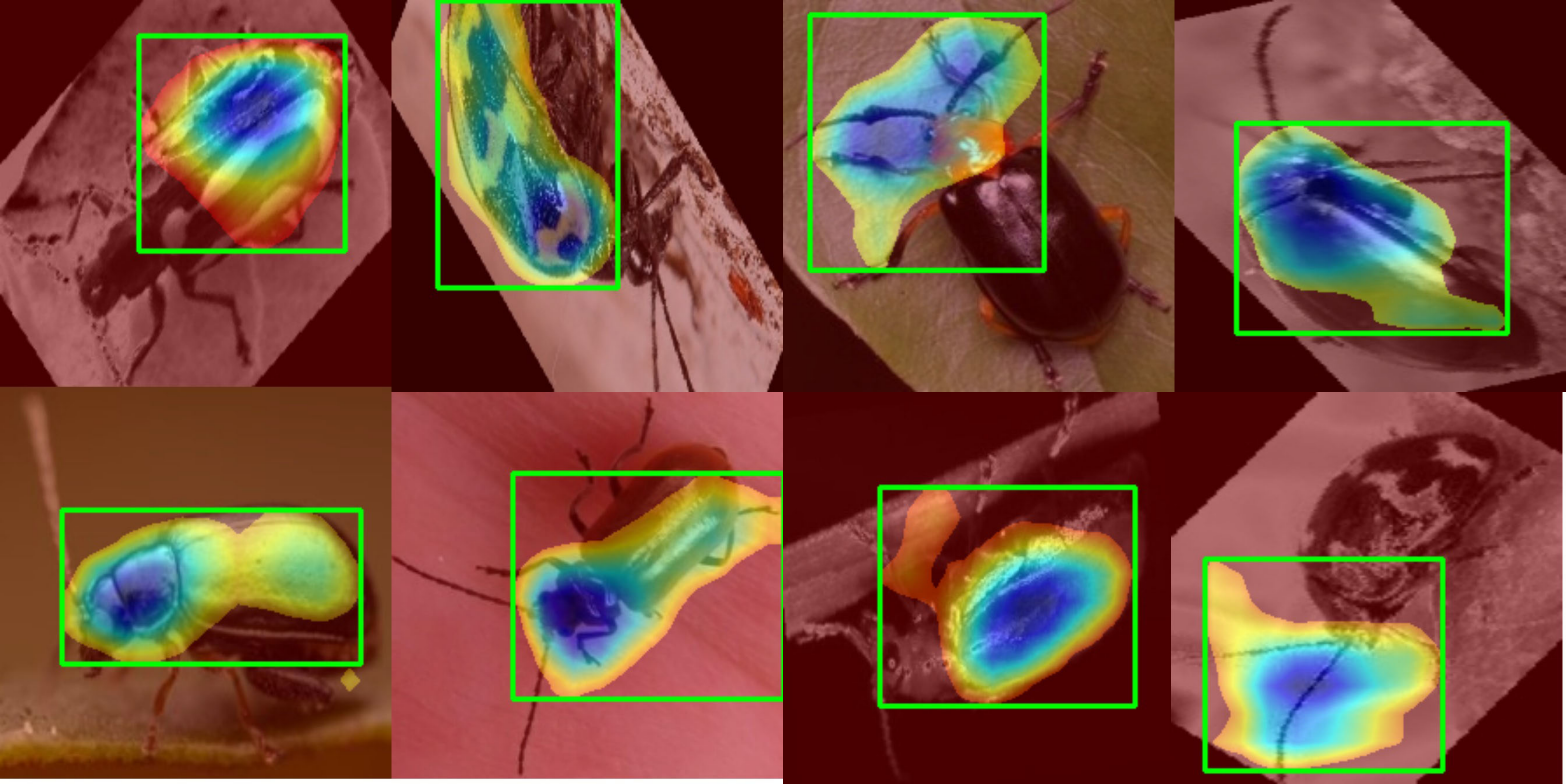
Grad-CAM visualization demonstrating robust background suppression across various specimens. The green boxes indicate the primary regions of interest where the model identifies key taxonomic features.

As demonstrated in **Figure 6**, the model exhibits a robust background suppression capability, which is a critical indicator of its decision-making reliability. In the representative samples shown, the beetle specimens are often situated against complex backgrounds, including textured leaves, soil, or irregular collection environments. However, the Grad-CAM heatmaps (indicated by the regions within the green bounding boxes) consistently show that the model effectively filters out these non-taxonomic distractions. Even when the insect is positioned on a highly textured leaf or a variegated surface, the high-activation zones (warm-colored regions) remain strictly confined to the biological boundaries of the beetle.

This selective focus confirms that the MVBeetle framework has successfully learned to decouple the target specimen from its acquisition environment. By centering its attention on stable phenotypic markers—such as the distinctive elytral patterns and the metafemoral spring region characteristic of flea beetles—the model achieves a taxonomic focus that aligns with expert identification protocols. The clear diagonal dominance in the confusion matrix is thus supported by this interpretability analysis, ensuring that the high classification accuracy is derived from genuine morphological differentiation rather than spurious correlations with background elements.

#### 3.3.1 Quantitative analysis of activation maps

**Figure 7:**
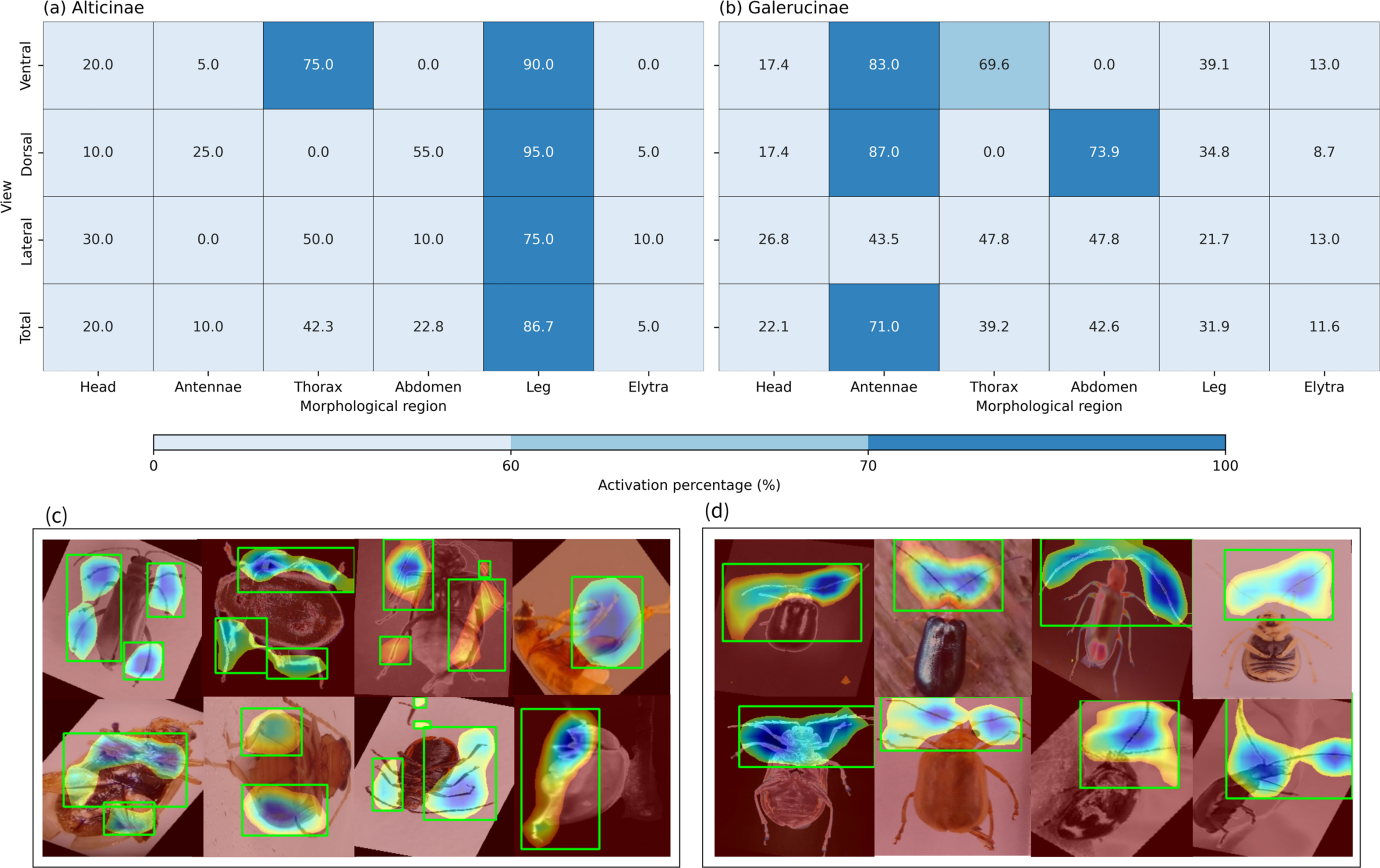
Grad-CAM interpretability analysis of Alticinae and Galerucinae. **(a)** Alticinae activation heatmap, showing a strong focus on leg features across all views; **(b)** Galerucinae activation heatmap, highlighting the importance of antennae and thoracic regions; **(c)** Saliency maps for Alticinae, illustrating the precise localization of hind legs; **(d)** Saliency maps for Galerucinae, showing the model’s focus on antennae.

The statistical distribution of these regional activations, illustrated in **Figures 7a, b**, reveals clear differences in the model’s attention patterns for each subfamily. For Alticinae, the model shows an overwhelming focus on the Leg region, with a total activation rate of 86.7%. This attention is most intense in the Dorsal view (95.0%) and Ventral view (90.0%). Notably, in the Ventral view, the Thorax also triggers a high response (75.0%). This specific focus makes biological sense, as these areas house the powerful muscles and spring organ that enable flea beetles to jump.

In contrast, the diagnostic focus for Galerucinae shifts toward the antennae and abdomen. The data indicates that antennae are a key feature, exhibiting an activation rate of 71.0% that peaks at 87.0% in the dorsal view. Furthermore, the MVBeetle model pays close attention to abdominal shapes in the dorsal view (73.9%) and thoracic textures in the ventral view (69.6%). These patterns suggest that the model identifies Galerucinae by evaluating overall body proportions and surface textures.

Overall, the correspondence between the model’s attentional hotspots and diagnostic traits used by human experts indicates that MVBeetle successfully captures the essential morphological characteristics of these beetles. Reference boundaries for the principal morphological parts were manually annotated on representative specimens to ensure that these regional activations were accurately mapped to specific anatomical structures. The annotation process was independently performed by two researchers with expertise in entomology, guaranteeing biological accuracy. Inter-observer reliability was assessed using Cohen’s Kappa coefficient, yielding a score of κ=0.92, which indicates near-perfect agreement between annotators and provides a robust ground-truth framework for interpreting the Grad-CAM activations.

#### 3.3.2 Spatial saliency maps and anatomical correspondence

The spatial heatmaps generated through Grad-CAM, as displayed in **Figures 7c, d**, provide direct visual evidence of the anatomical structures driving the classification process. For Alticinae **Figure 7c**, the high-intensity activation zones—indicated by the concentrated red and yellow regions—are predominantly localized on the hypertrophied hind femora. This localization confirms that the model successfully identified the metafemoral spring, which is the most distinctive evolutionary trait and jumping apparatus of flea beetles. The saliency mapping also extends to the junction between the hind legs and the abdomen, capturing the complex mechanical integration of the jumping mechanism.

In contrast, the heatmaps for Galerucinae in **Figure 7d** activation zones primarily target the antennae, especially the antennal bases and flagellomeres. The attention zones are primarily localized on the antennae, with a specific focus on the antennal bases and the flagellomeres. The MVBeetle model utilizes the segmental ratios and the specific morphological structure of the antennae to perform classification, this indicates that the model relies heavily on sensory-associated morphological traits for classification. This prioritization of sensory-based structural patterns contrasts sharply with the focus on “dynamic” locomotory structures, such as the hind femora, prioritized during Alticinae identification. Overall, these results demonstrate that MVBeetle distinguishes subfamilies by focusing on key anatomical markers consistent with those used by human taxonomists.

#### 3.3.3 Multi-perspective synergistic response

The multi-view integration strategy effectively captures complementary features that are often obscured or incomplete when viewed from a single perspective. Instead of relying on a dominant but potentially biased angle, the model constructs a comprehensive diagnostic profile by balancing information across three anatomical planes. The Dorsal View captures the specimen’s primary posture and head-to-antennae orientation, while the Lateral View provides critical depth information—such as the distinctive bulge of the metafemoral spring in Alticinae. Furthermore, the Ventral View adds fine-grained details of the ventral sclerites and leg junctions that remain invisible from other angles.

In summary, the Grad-CAM results confirm that the MVBeetle model accurately targets diagnostic morphological structures with high taxonomical value rather than superficial textures. This transition from recognizing general silhouettes to focusing on specific anatomical organs explains the high diagonal dominance achieved in the confusion matrix (**Figure 5**). By integrating these localized responses into a synergistic whole, the model effectively mimics the multi-angle examination process traditionally used by entomologists. Ultimately, these findings demonstrate that the multi-view approach provides not only superior accuracy but also a level of biological explainability that is essential for the future of digital entomological research.

## 4 Discussion

The classification performance of MVBeetle demonstrates the clear advantage of multi-view integration for addressing fine-grained taxonomic challenges in Chrysomelidae. By jointly exploiting dorsal, lateral, and ventral information, the model reconstructs a more complete morphological representation than single-view approaches, effectively reducing information loss caused by viewpoint bias, occlusion, or incomplete feature exposure. Many diagnostic characters in flea beetles and leaf beetles—such as hind femoral hypertrophy, body profile, and abdominal configuration—are spatially distributed across different anatomical planes, and their separation in single-view settings inevitably constrains recognition accuracy. The multi-view fusion strategy adopted here synthesizes complementary cues across perspectives, resulting in improved robustness and generalization, consistent with broader findings that multi-view learning outperforms single-view schemes in biological image analysis (Seeland and Mäder, 2021; Li et al., 2024).

Beyond performance improvements, attention-based interpretability analysis indicates that MVBeetle captures biologically meaningful diagnostic traits that align closely with established taxonomic knowledge in Chrysomelidae. For Alticinae, the model’s attention is predominantly localized on the hind femora and leg joints—structures directly associated with the hypertrophied metafemoral spring and jumping adaptations (Ge et al., 2010; Nadein and Betz, 2016). In contrast, the emphasis in Galerucinae shifts toward antennae and dorsal surface textures, reflecting the taxonomic importance of sensory structures and external phenotypic variation in leaf beetles (Duckett et al., 2004; Vig, 2004). These patterns suggest that the attention mechanism implicitly encodes functional distinctions between the two subfamilies, grounding classification decisions in relevant morphology rather than spurious background cues.

Previous studies have shown that transformer-based models such as Vision Transformers (ViTs) perform well in learning global representations when trained on large-scale datasets (Dosovitskiy et al., 2020; Liu et al., 2021). In contrast, convolutional neural networks (CNNs) possess stronger locality inductive biases and have demonstrated strong performance in learning fine-grained local features, particularly under relatively limited training data (Raghu et al., 2021). Therefore, considering the limited dataset size and the importance of subtle morphological traits in this study, CNN-based architectures were adopted, while future work will explore comparisons with transformer-based and hybrid architectures.

Beyond architectural considerations, a primary challenge lies in the inherent difficulty of multi-view data acquisition, particularly for the ventral perspective. In field conditions (*in situ*), ventral views are rarely available because insects are typically attached to substrates such as leaves or bark, which naturally obscure their underside. Even under laboratory conditions, capturing high-quality ventral images of mounted specimens is labor-intensive and may risk damaging fragile biological structures during repositioning. This practical constraint contributes to the relatively limited dataset size, which remains a potential limitation for model generalization. Specifically, the limited number of specimens per taxon may reduce the model’s ability to generalize to rare species or to capture substantial intra-species morphological variation across different geographical populations.

Furthermore, the interpretability afforded by Grad-CAM remains restricted to externally visible features. Internal diagnostic structures, such as genitalia or internal musculature, cannot be resolved without dissection or microscopy, limiting the capacity of image-based deep learning models to fully substitute traditional taxonomic practice. Consequently, MVBeetle should be regarded as a complementary tool for rapid screening, pest monitoring, and large-scale biodiversity surveys rather than a replacement for classical identification. Future work may explore generative data augmentation or advanced robotic imaging to bridge the gap between laboratory-grade multi-view data and field-based observations. Nonetheless, the current framework provides a scalable solution for chrysomelid identification, consistent with recent studies highlighting both the potential and interpretability limitations of deep learning in biological classification (Li et al., 2024).

## 5 Conclusion

This study proposes MVBeetle, a multi-view fusion deep learning model designed to address the long-standing challenges of high morphological similarity and low identification efficiency in Chrysomelidae, a family containing numerous economically significant agricultural pests. By integrating dorsal, lateral, and ventral images through a shared-weight encoder coupled with a self-adaptive view attention mechanism, the model dynamically prioritizes the most informative perspectives for each specimen. This architecture enables the capture of complementary diagnostic traits that are frequently obscured in traditional single-view analyses.

Experimental results demonstrate that MVBeetle achieves a state-of-the-art classification accuracy of 94.44% ± 0.41%, significantly outperforming baselines. Notably, the model exhibits exceptional taxonomic stability, with minimal cross-subfamily misclassification between Alticinae and Galerucinae, ensuring high reliability for high-level taxonomic screening. Grad-CAM visualizations confirm that attention allocation and predictions are guided by biologically meaningful features—such as the hypertrophied hind femora and antennal–thoracic structures—providing interpretability for taxonomic applications”.

While the current framework provides a robust and scalable foundation for automated beetle identification, future research will focus on key strategies to address current data constraints. These include the integration of three-dimensional (3D) reconstruction to capture a continuous morphological space, the implementation of generative data augmentation to alleviate the scarcity of multi-view training samples, and the optimization of the model for edge-device deployment to support real-time, in-field pest surveillance and quarantine inspection. Overall, MVBeetle represents a significant advancement in intelligent pest monitoring and large-scale biodiversity assessment.

## Data Availability

The raw data supporting the conclusions of this article will be made available by the authors, without undue reservation.
