# Supplementary material for: MVBeetle: an interpretable multi-view deep learning model for fine-grained classification of Galerucinae and Alticinae (Coleoptera: Chrysomelidae)
Source: Front Plant Sci. 2026 Apr 7;17:1798135. doi: 10.3389/fpls.2026.1798135 (PMC13095795; doi:10.3389/fpls.2026.1798135)
Supplement: Supplementary file 1 [file Table1.docx]

Supplementary Material

**Supplementary Table S1. Imaging conditions and camera settings for standardized three-view macro-photography**

| **Parameter** | **Setting** |
| --- | --- |
| Imaging protocol | Standardized three-view acquisition (dorsal, lateral, ventral) |
| Camera body | Canon EOS 850D DSLR |
| Lens | Canon EF 100 mm f/2.8L IS USM macro lens (1:1 magnification) |
| Illumination | Dual-sided LED cold light sources (5500 K, flicker-free, CRI > 95) |
| Background | Diffuse white background panel (reflectance > 98%) |
| Specimen support | Precision pan–tilt stage with fine-adjustment specimen holder |
| Exposure mode | Manual (M) |
| Shutter speed | 1/60 s |
| Aperture | f/8–f/11 (optimal sharpness range) |
| ISO sensitivity | 400 (primary) or 1250 (low-light compensation) |
| Exposure compensation | 0 EV |
| Flash | Disabled |
| Focus method | Manual focus with 10× live-view magnification |
| Image resolution | 4000 × 6000 pixels (24 MP) |
| File format | RAW (CR3), lossless post-processing, exported as JPEG (quality = 100) |

**Supplementary Table S2**. **Biological Rationale for Augmentation Parameters**

| Parameter | Operations | Biological/Technical Rationale |
| --- | --- | --- |
| Rotation | ±45° (5° increments) | Simulates variations in field observation angles |
| Flipping | Horizontal/Vertical | Preserves inherent bilateral symmetry of insects |
| Solarization | Threshold 128–255 | Simulates strong backlighting in the field |
| Gaussian Noise | σ = 0.01–0.05 | Simulates camera sensor noise |
| Light/Contrast | ±30% | Simulates uneven natural illumination |
| Blurring | Kernel size 1–3 px | Simulates handheld motion blur **or** defocusing |

**Supplementary Table S3. Training hyperparameters and computational environment specifications.**

| **Category** | **Parameter** | **Value / Setting** |
| --- | --- | --- |
| Hardware Environment | CPU | x86_64 (10 Physical Cores) |
|  | GPU | NVIDIA GeForce RTX 3090 (24GB VRAM) |
| Software Environment | Operating System | Linux 5.19.0-nvidia |
|  | CUDA / cuDNN | 12.1 / 9.1.0 |
|  | Python Version | 3.12.2 |
|  | Deep Learning Framework | PyTorch 2.5.1 |
|  | Core Libraries | OpenCV 4.12.0, Scikit-learn 1.6.1 |
| Experimental Control | Random Seeds | {42, 100, 2024, 7, 99} |
|  | Number of Runs | 5 |
| Training Settings | Batch Size | 64 |
|  | Maximum Epochs | 20 |
|  | Early Stopping Patience | 5 epochs |
| Optimization | Optimizer | Adam |
|  | Initial Learning Rate | 1 × 10⁻³ |
|  | Learning Rate Scheduler | StepLR |
|  | Step Size | 5 |
|  | Gamma | 0.1 |
|  | Weight Decay (L2) | 1 × 10⁻³ |
|  | Loss Function | Cross-Entropy Loss |
| Data Augmentation | Input Resolution | 224 × 224 |
|  | Augmentation Techniques | Rotation, Color Jitter, Affine, Grayscale |
|  | View Consistency | Synchronized augmentation across views |
| Visualization | Grad-CAM Threshold | Top 20% intensity |

**Supplementary Table S4.** **Summary of the multi-view Chrysomelidae image dataset**

| **Category** | Metric | Value | Description |
| --- | --- | --- | --- |
| Taxonomic scope | Species (subfamilies) | 43 (2) | 20 Alticinae + 23 Galerucinae |
| Original dataset | Total images | 3,342 | 1,796 raw + 1,546 GBIF images |
| Augmented dataset | Total images | 12,968 | ~3.9× augmentation |
|  | Avg. images per species | ~279 | After augmentation |
| View-wise distribution | Front (dorsal) | 4,538 | Head, thorax, and elytra |
|  | Side (lateral) | 3,916 | Hind femora and body profile |
|  | Bottom (ventral) | 4,514 | Ventrites and coxal structures |
| Multi-view structure | Multi-view groups | 4,278 | ≥2 views per specimen |
|  | Complete tri-view groups | 3,624 | Front + side + bottom |

**Supplementary Table S5.** **Summary of the multi-view Chrysomelidae image dataset**

| Class ID | Species Name | Dorsal Images | Lateral Images | Ventral Images | Total |
| --- | --- | --- | --- | --- | --- |
| S1 | **Agasicles hygrophila** | **16** | **15** | **15** | **46** |
| S2 | **Agetocera deformicornis** | **16** | **13** | **20** | **49** |
| S3 | **Altica sp** | **17** | **14** | **14** | **45** |
| S4 | **Aphthona chinensis** | **14** | **19** | **11** | **44** |
| S5 | **Aphthona sp** | **42** | **38** | **31** | **111** |
| S6 | **Aplosonyx yunlongensis** | **10** | **11** | **10** | **31** |
| S7 | **Argopus sp** | **21** | **0** | **20** | **41** |
| S8 | **Atrachya bipartita** | **29** | **25** | **10** | **64** |
| S9 | **Atrachya menetriesi** | **21** | **0** | **20** | **41** |
| S10 | **Atrachya pedestris** | **11** | **13** | **14** | **38** |
| S11 | **Aulacophora indica** | **26** | **29** | **25** | **80** |
| S12 | **Brachyphora nigrovittata** | **17** | **0** | **14** | **31** |
| S13 | **Charaea flaviventris** | **15** | **11** | **11** | **37** |
| S14 | **Charaea sp** | **16** | **18** | **26** | **60** |
| S15 | **Chrysomeloidea sp (Galerucinae)** | **28** | **0** | **23** | **51** |
| S16 | **Dercetina sp** | **55** | **49** | **56** | **160** |
| S17 | **Fleutiauxia armata** | **62** | **28** | **20** | **110** |
| S18 | **Gallerucida bifasciata** | **43** | **45** | **39** | **127** |
| S19 | **Hemipyxis flavipennis** | **28** | **27** | **24** | **79** |
| S20 | **Hemipyxis plagioderoides** | **15** | **15** | **16** | **46** |
| S21 | **Hermaeophaga sp** | **29** | **28** | **27** | **84** |
| S22 | **Hoplasoma sp** | **23** | **21** | **22** | **66** |
| S23 | **Hoplasoma unicolor** | **40** | **39** | **40** | **119** |
| S24 | **Hoplosaenidea bicolor** | **27** | **27** | **26** | **80** |
| S25 | **Longitarsus candidulus** | **28** | **0** | **29** | **57** |
| S26 | **Longitarsus cf. succineus** | **30** | **29** | **29** | **88** |
| S27 | **Longitarsus ochroleucus** | **32** | **28** | **29** | **89** |
| S28 | **Longitarsus salvae** | **11** | **28** | **29** | **68** |
| S29 | **Longitarsus tabidus** | **23** | **28** | **5** | **56** |
| S30 | **Macrima sp** | **28** | **28** | **25** | **81** |
| S31 | **Monolepta laticornis** | **22** | **0** | **21** | **43** |
| S32 | **Monolepta sp** | **25** | **36** | **25** | **86** |
| S33 | **Morphosphaera japonica** | **23** | **24** | **33** | **80** |
| S34 | **Neocrepidodera melanostoma** | **44** | **36** | **44** | **124** |
| S35 | **Nisotra gemella (Erichson)** | **44** | **44** | **44** | **132** |
| S36 | **Paleosepharia sp** | **17** | **20** | **21** | **58** |
| S37 | **Paragetocera favipes Chen** | **25** | **26** | **23** | **74** |
| S38 | **Paridea anguliconis** | **80** | **91** | **34** | **205** |
| S39 | **Phyllotreta striolata** | **30** | **44** | **39** | **113** |
| S40 | **Podontia lutea** | **17** | **0** | **16** | **33** |
| S41 | **Pseudoargopus sp** | **10** | **0** | **10** | **20** |
| S42 | **Psylliodes cyanescens** | **31** | **42** | **40** | **113** |
| S43 | **Sangariola fortunei** | **40** | **36** | **38** | **114** |

**Supplementary Table 6. Statistical comparison of single-view and multi-view models across four CNN backbones**

| **Model** | **Comparison** | **McNemar Test (p)** | **Paired t-test (p)** | **Wilcoxon Test (p)** |
| --- | --- | --- | --- | --- |
| **ResNet18** | **Multi-View vs Baseline** | **<0.001** | **0.002** | **0.004** |
|  | **Multi-View vs Concat** | **0.012** | **0.010** | **0.062** |
| **ResNet50** | **Multi-View vs Baseline** | **<0.001** | **0.003** | **0.006** |
|  | **Multi-View vs Concat** | **0.004** | **0.007** | **0.010** |
| **VGG16** | **Multi-View vs Baseline** | **<0.001** | **4.79e-6** | **0.0625** |
|  | **Multi-View vs Concat** | **0.002** | **0.00134** | **0.0625** |
| **MobileNetV2** | **Multi-View vs Baseline** | **0.012** | **0.018** | **0.021** |
|  | **Multi-View vs Concat** | **0.034** | **0.041** | **0.048** |
